# Characterization of aminopeptidase encoding gene *anp-1* and its association with development in *Caenorhabditis elegans*

**DOI:** 10.7717/peerj.7944

**Published:** 2019-11-12

**Authors:** Shanchun Su, Baoliang Pan, Yanxin Hu, Ming Wang

**Affiliations:** 1 Key Laboratory of Animal Epidemiology and Zoonosis of Ministry of Agriculture, College of Veterinary Medicine, China Agricultural University, Beijing, Beijing, China; 2 Institute of Anesthesiology and Pain (IAP), Taihe Hospital, Shiyan, Hubei, China

**Keywords:** *Caenorhabditis elegans*, Aminopeptidases, Lifespan, Body size

## Abstract

**Background:**

Aminopeptidases play important roles in various biological processes in nematodes including growth, development and reproduction. Although the aminopeptidases have been shown to regulate reproduction in *Caenorhabditis elegans* (*C. elegans*), the role of aminopeptidases in development and aging has not been reported. This study focused on the function of aminopeptidase AlaNyl aminopeptidase 1 (ANP-1) on development in *C. elegans*.

**Methods:**

In the present study, we reported the identification of ANP-1 in *C. elegans* along with sequence analysis and its functional expression and characterization. The phenotype changes were observed when *anp-1* mutated. Then, differential expression genes (DEGs) between wild type strain (N2) and *anp-1* deletion strain (RB804) were identified using transcriptome sequencing method. Finally, DEGs were verified by qRT-PCR assay.

**Results:**

Our observations suggested that *anp-1* mutation induced small body size in the L4/young adult stage of *C. elegans*, however, there was no difference between N2 and RB804 in adult stage. Moreover, deletion of *anp-1* resulted in shortening lifespan and laying fewer eggs. DEGs (184 genes) were observed between N2 groups and RB804 groups by transcriptome sequencing. According to GO annotations and KEGG enrichment analysis, these DEGs play vital roles in development regulation in *C. elegans*. These data demonstrate ANP-1 participates in development and aging of *C. elegans* and will considerably contribute to the existing knowledge of aminopeptidase function in *C. elegans*.

## Introduction

Aminopeptidases are protease enzymes present in all mammals, insects, plants, and bacteria (Drinkwater et al., 2017). Aminopeptidases participate in a diverse set of physiological and biological processes (BP) such as small peptide hormone modification, protein trafficking, cell cycle progression, signal transduction, antigen presentation and autophagy (Constam et al., 1995; Matsui, Fowler & Walling, 2006; Tobler et al., 1997). Regarding their active sites, substrate specificity, and other molecular properties, most aminopeptidases are characterized as metalloenzymes (Mucha et al., 2010; Rawlings, Barrett & Bateman, 2012; Rawlings, Tolle & Barrett, 2004). For example, M1 family of aminopeptidases are characterized by a conserved zinc-dependent site, HEXXH-(X)_18_-E motif, as well as an exopeptidase motif “GXMEN” (Iturrioz et al., 2001; Laustsen, Vang & Kristensen, 2001). Puromycin-sensitive aminopeptidase (PSA) is a member of the M1 metallo-aminopeptidase family and was first found in rat brain tissue (McLellan et al., 1988). PSA possesses conserved motifs that are characteristic of the M1 aminopeptidase family (Laustsen, Vang & Kristensen, 2001). *Psa* orthologs exist in the genome of every multicellular organism examined (Hersh, 1981; Hui, Wang & Lajtha, 1983). In mice, worms and other model organisms, PSA has been found to play essential roles in growth and behavior (Osada, Sakaki & Takeuchi, 1999). Mutations in *psa* orthologs result in meiotic errors and reduced embryonic viability (Osada, Sakaki & Takeuchi, 1999; Pradillo et al., 2007; Sanchez-Moran et al., 2004; Schulz, Perezgasga & Fuller, 2001). Another PSA called puromycin-sensitive aminopeptidase-1 (PAM-1), an ortholog of PSA, is the first M1 aminopeptidase that was characterized in detail in *Caenorhabditis elegans* (*C. elegans*). Homozygous *pam-1* mutations produce analogous phenotypes as *psa* (Lyczak et al., 2006). In *C. elegans*, 16 paralogs are defined as M1 aminopeptidases including PAM-1. RNA interference (RNAi) assays suggest that PAM-1 is not the only aminopeptidase associated with reproductive functions in *C. elegans*. RNAi-mediated inhibition in the aminopeptidases Y67D8C.9, T16G12.1, and T12E12.6 decreases brood size and oocyte maturation of *C. elegans* in distinct pathways, whereas, paralogs C42C1.11, R03G8.4, and ZC416.6 are not involved in *C. elegans* reproductive functions (Althoff, Flick & Trzepacz, 2014). Furthermore, PAM-1 and its orthologs (C42C1.11, R03G8.4, and ZC416.6) may have undiagnosed functions as they do have recognized roles in neuronal tissues (Kamath et al., 2003; Sonnichsen et al., 2005).

AlaNyl aminopeptidase 1 (ANP-1) is a paralog of PAM-1, it is expressed in the pharynx, nervous system, intestine, and excretory cells of *C. elegans* (https://www.wormbase.org/species/c_elegans/gene/WBGene00011587#01-9g-3) (Hunt-Newbury et al., 2007). RNAi-mediated suppression of ANP-1 shows that it does not play an identifiable role in the reproductive mechanisms of *C. elegans* (Althoff, Flick & Trzepacz, 2014). However, little is known about the function of ANP-1 and other aminopeptidases in development and aging.

In this study, we cloned and expressed an ANP-1 aminopeptidase fragment and determined its enzymatic activity. We characterized phonetype changes of body size, brood size, and lifespan used a *anp-1* deficient strain. Differential expression genes (DEGs) were observed and validated between *anp-1* mutation strain and wild type strain. Our data suggest that *anp-1* encodes a membrane aminopeptidase that participates in the development and lifespan regulation in *C. elegans*. This study provides the first insight into the development and lifespan regulation of aminopeptidase in *C. elegans*.

## Materials and Methods

### cDNA synthesis and multiple alignments

*C. elegans* hermaphrodites (N2 strain) and *anp-1* deletion strain (RB804) were obtained from the Caenorhabditis Genetics Centre (CGC), University of Minnesota, USA. All *C. elegans* strains were cultivated on NGM plates at 20 °C (Stiernagle, 2006). Wild type worms were harvested with mixed stages and washed three times with cold 1×M9 buffer. After grinding in liquid nitrogen, total RNA was extracted from the clean worms using TRIZOL (Invitrogen, Carlsbad, CA, USA) as per manufacturer′s instructions. Then, cDNA was generated using the SuperScript™ III Reverse Transcriptase kit (Invitrogen, Carlsbad, CA, USA). There are two transcript isoforms of *anp-1* (WBGene00011587), *t07f10.1a* and *t07f10.1b*, only the longest isoform (*t07f10.1a*) was considered in the present study. The specific forward and reverse primers designed for *anp-1* were 5′-ATGGCCTCCGCCTACACTTATG-3′ and 5′-TTCAAAGGCTCTTCGAGATCTC-3′. A total of 30 cycles of polymerase chain reaction (PCR) were performed with the following protocol: 95 °C for 5 min, 94 °C for 30 s, 56 °C for 30 s, 72 °C for 3 min and 72 °C for 10 min. The PCR product was purified using the QIAquick® PCR Purification Kit (QIAGEN, Hilden, Germany), cloned into a pEASY-T1 plasmid (TransGen, Beijing, China) and named “pEASY-*anp*-1”. The recombinant vector was transformed into DH5α competent cells, from which clones were identified by colony PCR and sequenced for verification by company (The Beijing Genomics Institute (BGI), Shenzhen, China).

In this study, aminopeptidase sequence alignments were generated by CLUSTAL W (Larkin et al., 2007) and ESPript 3. A phylogenetic tree of the M1 aminopeptidase family (cd09601) was rendered using MEGA6 software and FigTree v1.4.3 (Dereeper et al., 2008). The transmembrane region and glycosylation residues were predicted using TMHMM Server v. 2.0 and NetOGlyc 4.0 Server, respectively. The amino acid sequence of ANP-1 was used for homology modeling to predict the three-dimensional (3D) model crystal structure. The 3D structure was checked by PROCHECK.

### Aminopeptidase activity assay and production of polyclonal antibody against recombinant ANP-1

The conserved M1 aminopeptidase sequence of ANP-1 was cloned into the pET28a vector and expressed as a recombinant protein (rANP-1) in the *Escherichia coli* (*E. coli*) strain BL21 (DE3) cells induced by 1 mM isopropyl β-D-1-thiogalactopyranoside (IPTG) for 4 h at 37 °C. The forward and reverse primers designed for ANP-1 carrying restriction enzyme sites for *BamHI* or *XhoI* (in italics) were 5′-*CGCGGATCC*ATCAGTTATCAATTGACAGTCA-3′ and 5′-*CCGCTCGAG*AGGATATCCCAGCTGTTTAG-3′, respectively. Briefly, bacterial lysate pellet was washed in Buffer A (2 M urea) and Buffer B (4 M urea) before being resuspended in Buffer C (8 M urea, 25 mM Na_2_HPO_4_, 50 mM Tris-HCl, 100 mM NaCl, 10% (v/v) glycerol, pH 7.5). Then the protein were purified using an Ni-NTA affinity column (GE Healthcare, Chicago, IL, USA). The affinity column was washed with 10 volumes of Buffer D (40 mM imidazole) and Buffer E (60 mM imidazole), and the fusion protein was eluted with Buffer F (8 M urea, 250 mM imidazole, at pH 7.5). The eluted protein was dialyzed against 1x PBS with a urea gradient (4 M, 2 M, 1 M and 0 M urea) and protein concentration was estimated by a BCA protein assay kit (Pierce, Appleton, WI, USA). The molecular weight of rANP-1 protein was confirmed by Coomassie-stained SDS-PAGE gels (12%) and western blotting. Then, the polyclonal antibody against rANP-1 was prepared according to the previous study (Munkhjargal et al., 2016).

Aminopeptidase activity and inhibitor sensitivity of rANP-1 were tested via standard assays using the leucine aminopeptidase (LAP) substrate L-leucine-p-nitroanilide (L-Leu-pNA; Sigma) as previously described (Roberts et al., 2013). Absorbance was measured at 405 nm using a Tecan Sunrise Microplate Reader.

### Proteins extraction and identification

Mix-stage N2 worms were maintained and harvested using liquid culture method (Zhou et al., 2014). Worms were stored at 80 °C until use. The membrane proteins were extracted following the manufacturer’s instructions of Mem-PER™ Plus Membrane Protein Extraction Kit (Thermo Fisher, Waltham, MA, USA). Then, the crude glycoproteins of *C. elegans* were extracted using ConA according to the previous study (Redmond, Geldhof & Knox, 2004). The membrane proteins and glycoproteins were confirmed by SDS-PAGE and western blot assays.

### Lifespan assay

The animals were synchronized by alkaline hypochlorite treatment (20 mL total volume with 1.25 mL of 5 M NaOH, 5 mL of NaClO and 13.75 mL of water). Synchronized eggs were incubated in sterilized M9 buffer for about 16–20 h at 20 °C (200 revolutions/min) until the L1 larval stage, and the L1 larvae were transferred to freshly prepared NGM plates for lifespan experiments.

At least 50 L1-stage worms were picked and transferred to 5-fluoro-20-deoxyuridine (FUDR)-NGM medium dishes and seeded with fresh bacteria for food. This was recorded as Day zero (Lezzerini et al., 2015). Surviving worms were removed every 24 h until all of the worms had died. A worm was considered dead when it did not respond to a gentle touch with a pick needle. Nematode lifespan was measured as the time till death for 50% of the worms. Experiments were repeated three times independently.

### Scoring body size and determining brood size

Two percent agarose glass pads were made according to the method described in Liang, Xiong & Savage-Dunn (2013). Briefly, 50 μL NaN_3_ (25 mM) was added to each agarose pad labeled with a unique color, about 50 live L4/ young adult worms or adult worms were anesthetized in this NaN_3_ solution and then coverslips were placed on the top. Images were captured and measured using a Zeiss stereoscope (Discovery v.12) with supporting software.

One synchronized L1-stage (see above) nematode was randomly picked from a correspondingly labeled NGM plate and cultivated into a young adult larva (about 48 h). The young adults were then shifted daily to new dishes before laying their first egg (Yu et al., 2015). Adult nematodes were moved every 6 h until they stopped laying eggs. The total number of eggs laid by individual hermaphrodites was calculated. Ten worms per strain were used.

### RNA extraction, library preparation, and illumina hiseq xten sequencing

Total RNA was extracted from the L4/young adult worms according to the approach described previously and genomic DNA was removed using DNase I (TAKARA). RNA-seq transcriptome librariy was prepared following TruSeq™ RNA sample preparation Kit from Illumina (San Diego, CA, USA) using 5 μg of total RNA. Libraries were size selected for cDNA target fragments of 200–300 bp on 2% Low Range Ultra Agarose followed by PCR amplified using Phusion DNA polymerase (NEB) for 15 cycles. After quantified by TBS380, paired-end RNA-seq sequencing library was sequenced with the Illumina HiSeq xten (2 × 150 bp read length) at Majorbio Bio-Pharm Technology Co., Ltd., Shanghai, China.

### Read mapping, differential expression analysis and functional enrichment

The raw paired end reads were trimmed and quality controlled by SeqPrep (https://github.com/jstjohn/SeqPrep) and Sickle (https://github.com/najoshi/sickle) with default parameters. Then clean reads were separately aligned to reference genome with orientation mode using TopHat (http://ccb.jhu.edu/software/tophat/index.shtml, version2.0.0) software (Trapnell, Pachter & Salzberg, 2009). To identify DEGs between two different samples, the expression level of each transcript was calculated according to the fragments per kilobase of exon per million mapped reads (FRKM) method. RSEM (http://deweylab.biostat.wisc.edu/rsem/) was used to quantify gene abundances (Li & Dewey, 2011). R statistical package software EdgeR (Empirical analysis of Digital Gene Expression in R, http://www.bioconductor.org/packages/2.12/bioc/html/edgeR.html) was utilized for differential expression analysis (Robinson, McCarthy & Smyth, 2010). In addition, functional-enrichment analysis including GO and KEGG were performed to identify which DEGs were significantly enriched in GO terms and metabolic pathways at Bonferroni-corrected *p*-value ≤ 0.05 compared with the whole-transcriptome background. GO functional enrichment and KEGG pathway analysis were carried out by Goatools (https://github.com/tanghaibao/Goatools) and KOBAS (http://kobas.cbi.pku.edu.cn/home.do) (Xie et al., 2011).

### RNA extraction and qRT-PCR analysis

A total of 500 ng RNA (from synchronized L4/ young adult worms) was used for qRT-PCR analysis and performed in triplicate with the TransScript II Green One-Step qRT-PCR SuperMix kit (TRANSGEN BIOTECH, Beijing, China) according to the manufacturer’s instructions. Thermal cycling and fluorescence detection were performed using a Roche LightCycler 96 System. The fold change of gene expression was calculated using the 2^−ΔΔCt^ method. The relative fold change of mRNA expression was normalized to the level of GAPDH expression. The primers designed for qRT-PCR are listed in Table S1.

### Statistical analyses

All data were represented as mean ± SEM. Student’s *t*-test was used to analyze brood size, body size, and relative mRNA expression levels between N2 strain and RB804 strain. Median survival days and *p*-values were calculated using the log-rank method (Mantel-Cox). All statistical analyses were conducted using GraphPad Prism 5.0. In all cases, the two-tailed *p*-value was calculated and *p* ≤ 0.05 was considered significant.

## Results

### Multiple alignments and 3D structure of ANP-1

**Figure 1:**
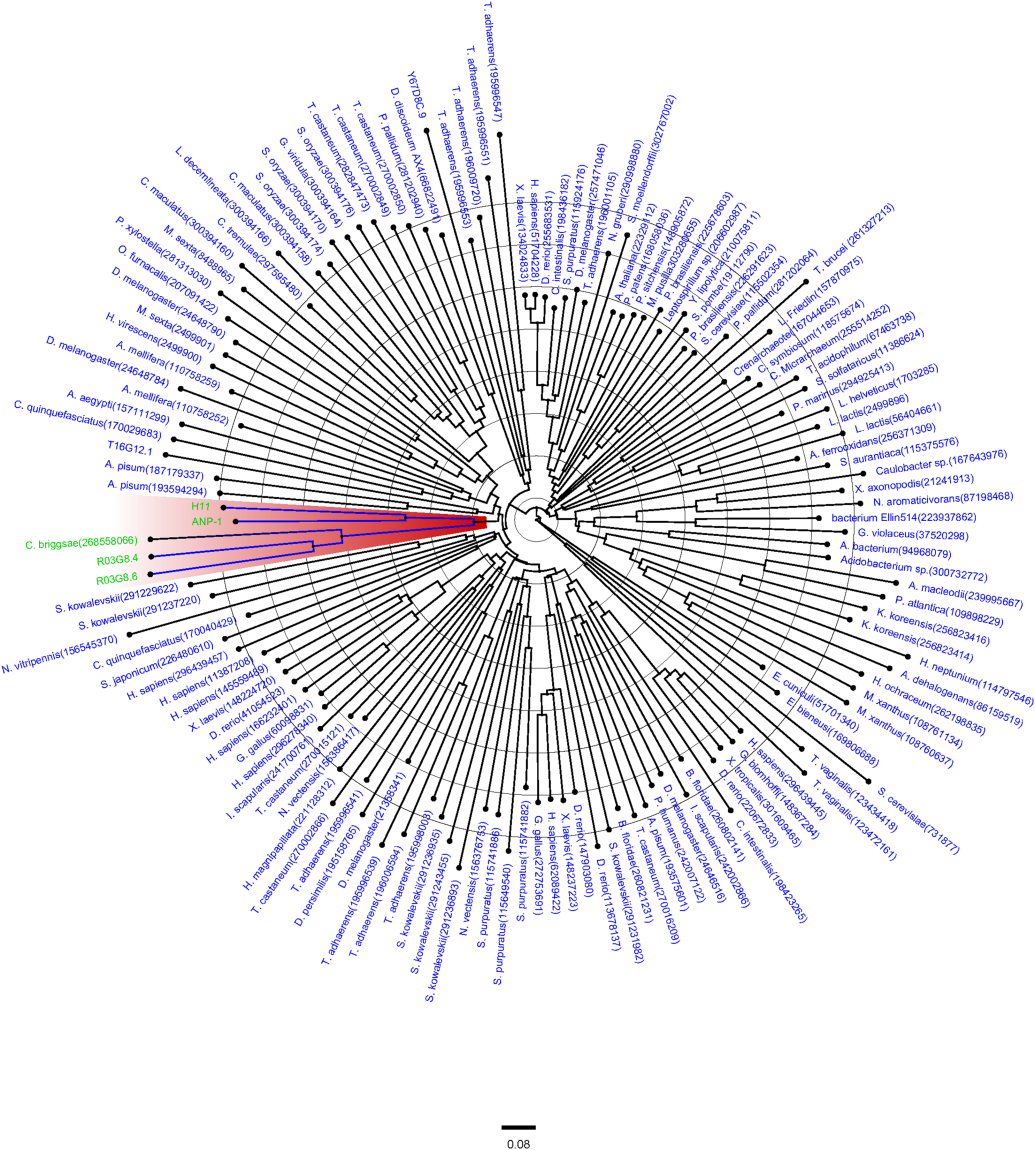
Phylogenetic relationships between members of the M1 aminopeptidase family (cd09601). Protein numbers and names of different species are given according to the NCBI website. The relationships of ANP-1 and its homologs (H11, R03G8.4 and R03G8.6) are labeled in green and highlighted in red. The calibration bar “0.08” is at the bottom of the figure.

To investigate the similarity of ANP-1 with other aminopeptidases, we cloned and sequenced the gene encoding *C. elegans* aminopeptidase ANP-1 (Fig. S1). The molecular weight (MW) of ANP-1 was about 110 kDa based on the translated property of coding sequence. Multiple alignment results showed that ANP-1 was a member of the M1 aminopeptidase family (Fig. S2). The signature functional motif “HEXXH-(X)_18_-E” and the exopeptidase motif “GXMEN” were conserved across all members of the M1 aminopeptidase family. PROTEIN BLAST result manifested that ANP-1 possessed M1 aminopeptidase N-2 (M1-APN-2) domain as well as an adipocyte-derived leucine aminopeptidase (ERAP1) functional domain. Sequence analysis revealed that ANP-1 had 42%, 33% and 34% identity with H11, R03G8.4 and R03G8.6, respectively. M1 aminopeptidase proteins from different species were analyzed based on the neighbor-joining (NJ) method. The phylogenetic relationship analysis of amino acid sequence data for H11, ANP-1, and their homologs (R03G8.4 and R03G8.6) from *C. elegans* showed strong support for a single clade (Fig. 1).

**Figure 2:**
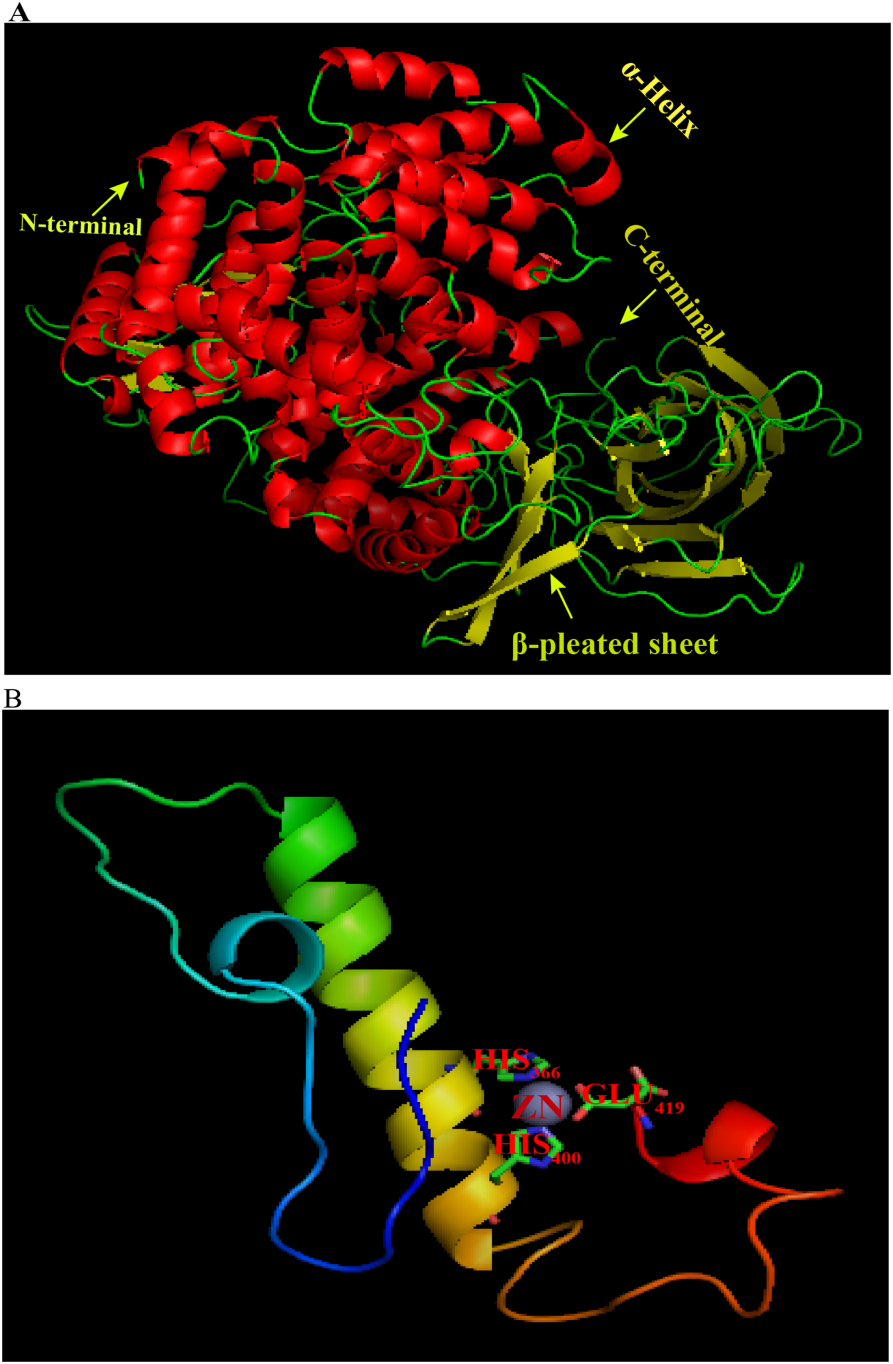
3D molecular model of ANP-1 based on the crystal structure of porcine 5lg6.1.A. (A) The α-helices and β-pleated sheets are indicated using arrows and the different colors denote different protein domains. (B) The gray sphere represents a zinc ion and the predicted zinc-binding residues are in stick representation.

Homology modeling of ANP-1 was conducted with SWISS-MODEL (Fig. 2A). The aminopeptidase N (APN) 5lg6.1.A was selected based on its identity (about 34%) to ANP-1. The 3D structure of ANP-1 consisted of 28 helices, 31 *β*-pleated sheets, and 12 random coil (Fig. S3). One zinc ion was bound in the active site of the protein and the zinc-binding residues (HIS_366_, HIS_400_, and GLU_419_) are represented with sticks (Fig. 2B). The 3D structure of ANP-1 protein was checked with PROCHECK for its stability and was found to be stable.

The asparagine-modified residues (GalNAc O-glycosylation sites) prediction result implied that ANP-1 was a transmembrane-glycopeptide protein. The feasible transmembrane helix of ANP-1 was from 29aa (amino acid, aa) to 51aa based on Hidden Markov Model (Fig. S2).

### Aminopeptidase activity of recombinant ANP-1

**Figure 3:**
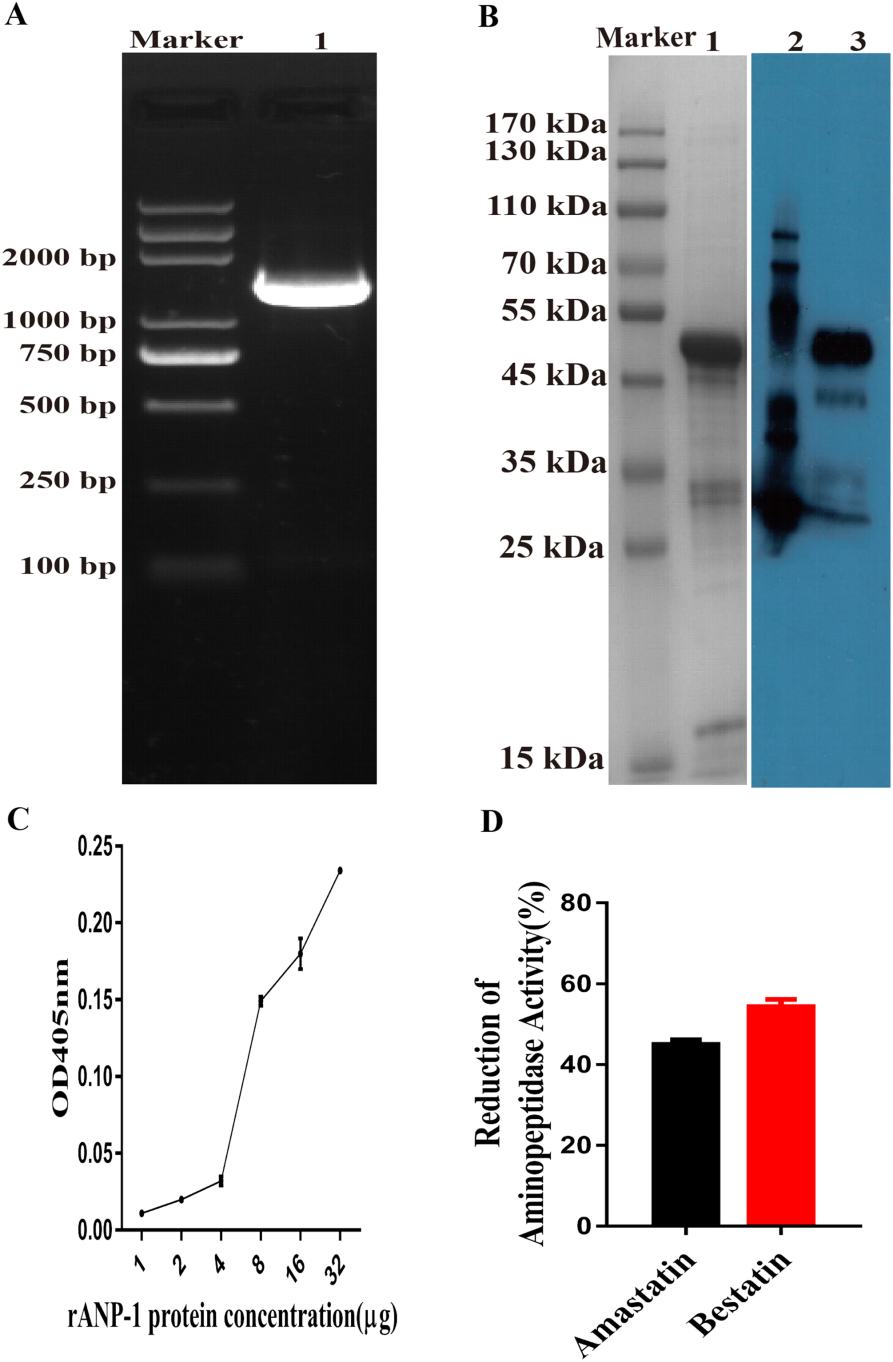
Aminopeptidase activity of rANP-1 protein. (A) Amplification of the *anp-1* gene aminopeptidase fragment by PCR using specific primers. Lane “M” contains the DL2000 Plus DNA marker and lane 1 shows the aminopeptidase fragment of *anp-1* gene (1401 bp). (B) Expression and purification of rANP-1 induced by IPTG. Lane “M” contains the protein marker, lane 1 shows the purified rANP-1 protein (52.7 kDa), lane 2 contains the EasySee Western Marker (TRANSGEN BIOTECH, Beijing, China), and lane 3 shows rANP-1 protein analysised by Western blot using anti-HIS tag monoclonal antibody (1:2000). (C) Aminopeptidase activity of rANP-1 protein was measured using L-Leu-pNA as substrate. (D) Aminopeptidase activity of rANP-1 was measured in the presence of the inhibitors amastatin or bestatin, and reduction (%) calculated relative to pre-incubation in buffer alone. Error bars represent standard errors.

The putative aminopeptidase fragment was amplified and cloned into a pET-28a plasmid (Fig. 3A). The rANP-1 was expressed in its insoluble form in the *E. coli* strain BL21 (DE3) induced by IPTG. The rANP-1 protein was purified and refolded before assessing by SDS-PAGE (12%) and western blotting. The results implied that we obtained rANP-1 protein and, the molecular weight of rANP-1 was about 52.7 kDa, which was consistent with the predicted result (Fig. 3B).

Aminopeptidase activity of rANP-1, measured by standard aminopeptidase assay, showed that rANP-1 retained the enzyme catalytic function. The enzymatic activity of rANP-1 was increased at higher concentrations (Fig. 3C). The aminopeptidase activity of rANP-1 was inhibited to a similar degree by the inhibitors amastatin and bestatin and the reduction rates of amastatin and bestatin were about 55% and 46%, respectively (Fig. 3D). Both the activity and inhibition assays suggested that the rANP-1 was refolded appropriately into an active protein.

### Proteins composition of membrane and ConA-binding fraction of *C. elegans*

**Figure 4:**
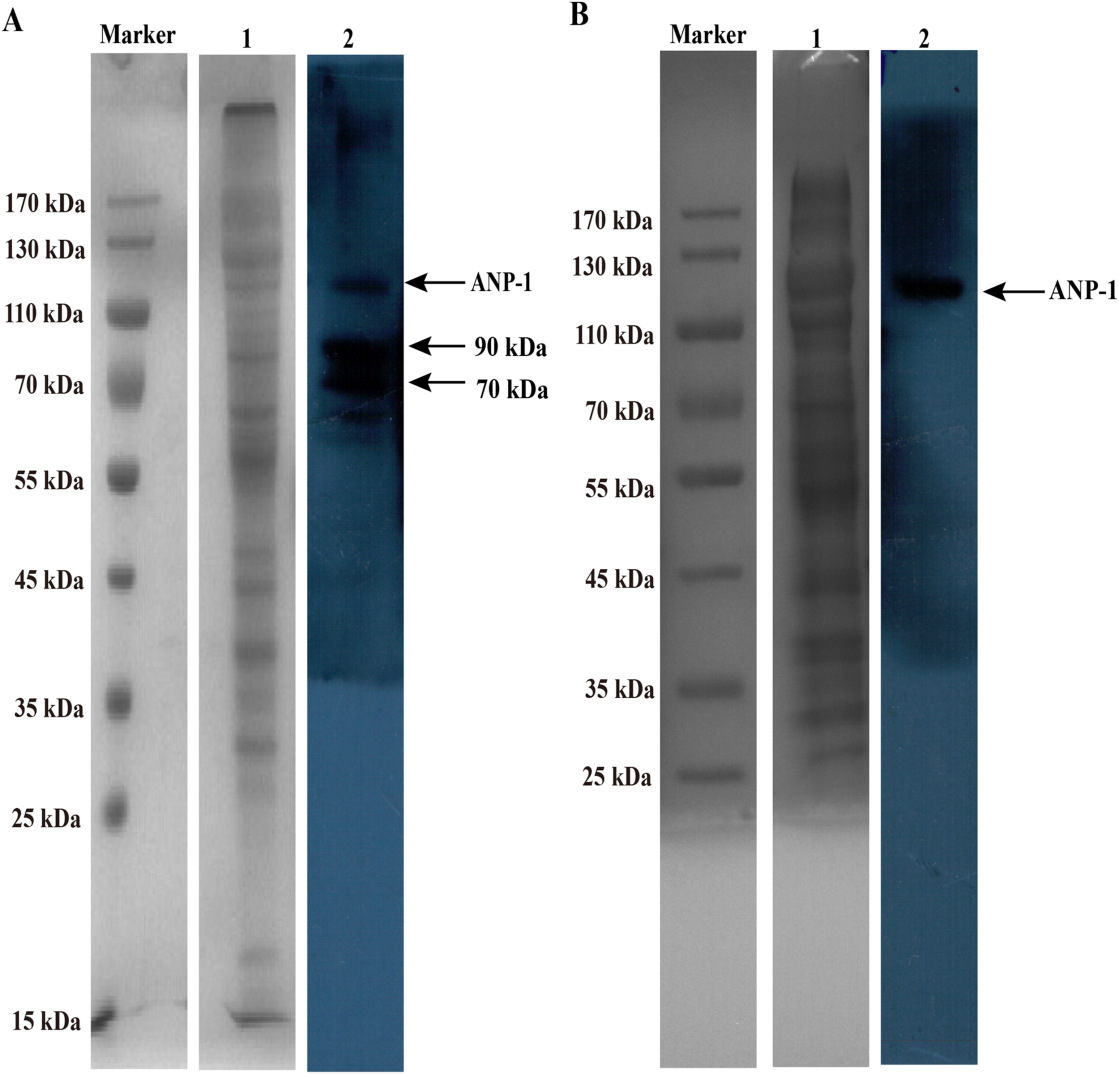
SDS-PAGE and western blot analysis of membrane and ConA-binding fraction of *C. elegans*. (A) Lane “M” contains the protein marker and lane 1 shows the membrane protein extracted from *C. elegans*. Lane 2 shows the proteins mainly reacted with anti-rANP-1 serum. Arrows indicate ANP-1 (about 110 kDa) and the other proteins’ bands. (B) Lane “M” and lane 1 show the protein marker and ConA-binding protein extracted from *C. elegans*, respectively. Lane 2 shows anti-rANP-1 serum only recognized ANP-1.

The membrane and ConA-binding peptides were separated under reducing SDS-PAGE conditions, ranging in size from approximately 15 kDa – >170 kDa (Fig. 4A, lane 1) and 25 kDa – >170 kDa (Fig. 4B, lane 1). Several of membrane proteins reacted with anti-rANP-1 serum, and the MW of those proteins were between 70 kDa and 130 kDa (Fig. 4A, lane 2). Western blot strip of *C. elegans* ConA-binding proteins were incubated with anti-rANP-1 serum. However, the antibody recognized only one band (Fig. 4B, lane 2), the band has the size of 110 kDa, which was consistent with the predicted MW of ANP-1.

### Deletion of *anp-1* decreased the lifespan and affected the body size and brood size in *C. elegans*

**Figure 5:**
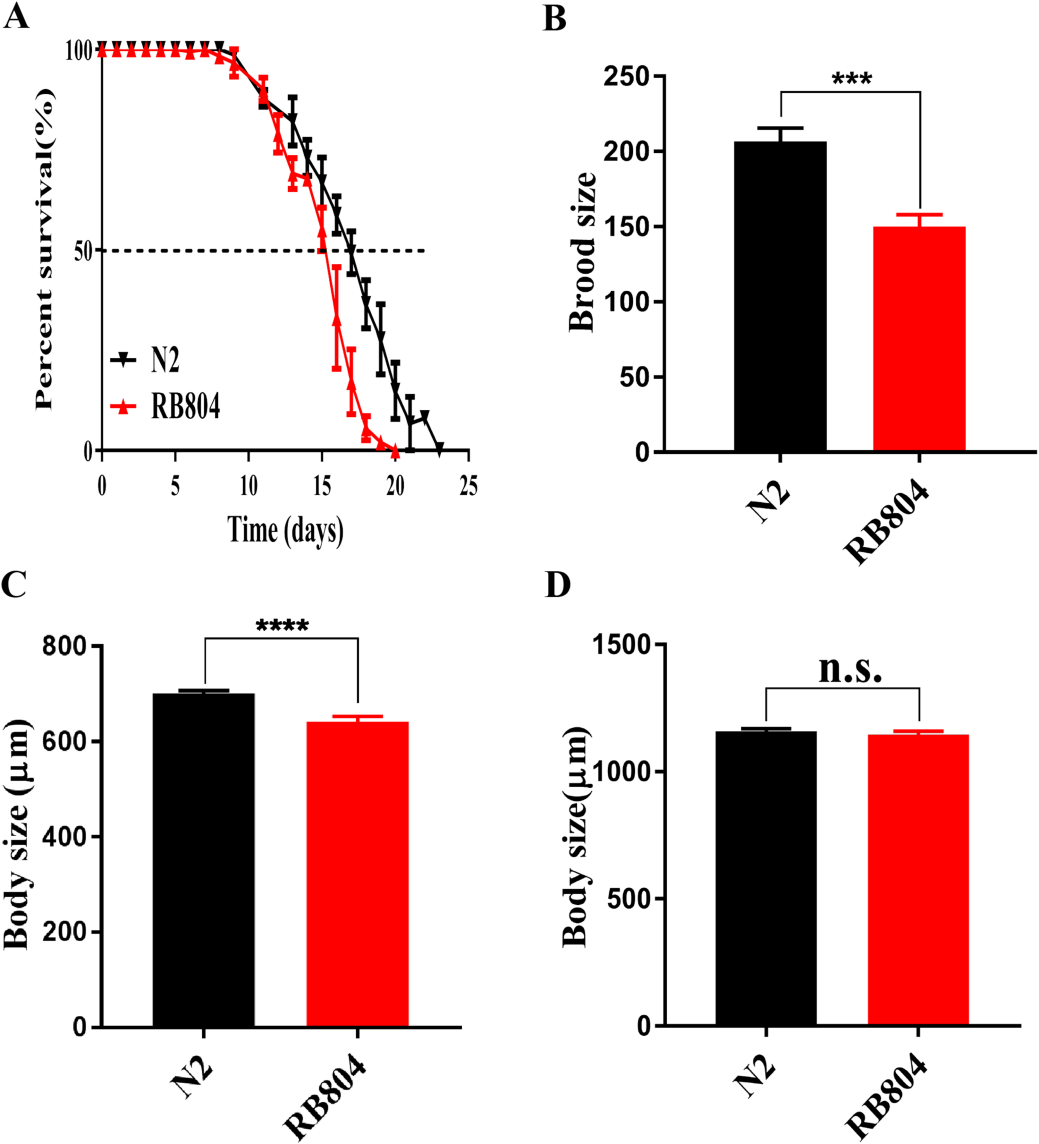
Mutation of *anp-1* gene induced phenotype changes between *C. elegans* N2 strain and RB804 strain. (A) The lifespan of N2 strain was significantly decreased compared to RB804 strains (*p* < 0.001). (B) RB804 animals produced fewer eggs compared with N2 animals. (C) and (D) Deletion of *anp-1* decreased the body size of L4/young adult worms rather than adult worms. Error bars represent standard errors and *** indicates a statistical significance *p* < 0.001.

As shown in picture 5, the median survival of N2 group and RB804 group were about 18 days and 16 days, respectively. Compared to the N2 strain, the *anp-1* mutation significantly decreased the lifespan of RB804 strain, *p* < 0.001 (Fig. 5A). Meanwhile, the body size of L4/young adult worms and adult worms was observed and measured as 701.2 ± 5.905 μm (N2 group, *n* = 50), 642.5 ± 10.66 μm (RB804 group, *n* = 48), 1158 ± 11.73 μm (N2 group, *n* = 50), and 1145 ± 14.25 μm (RB804 group, *n* = 49), which indicated that the body size of RB804 worms was significantly lower (*p* < 0.001) compared with the normal worms (Fig. 5C). However, there was no significant difference in adult body size of *C. elegans* when *anp-1* deleted (Fig. 5D). Representative images of animal body length were shown in Fig. S4. Furthermore, we found that knockout of *anp-1* gene significantly decreased total number of eggs of *C. elegans* (Fig. 5B), the brood size of N2 group and RB804 group were 206.7 ± 8.82 (*n* = 10) and 150 ± 7.95 (*n* = 10), respectively.

### DEGs analysis and functional annotation

**Figure 6:**
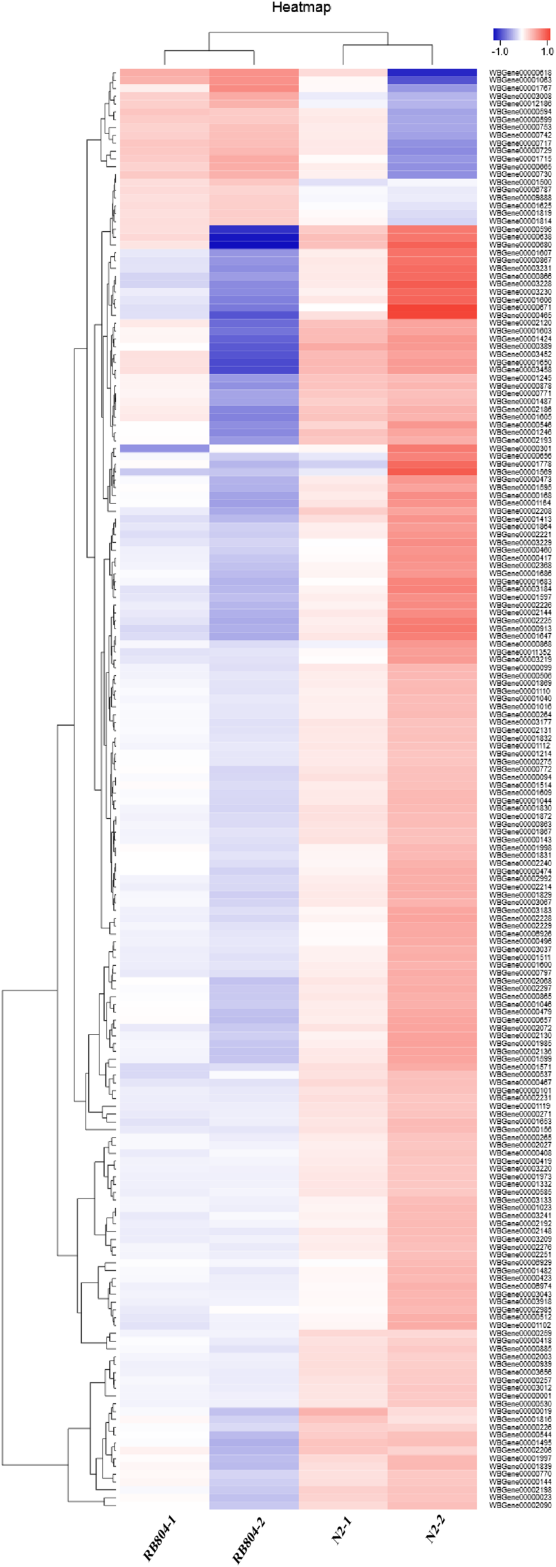
Hierarchical clustering of differential expression genes (DEGs) between N2 groups and RB804 groups. The color indicates the gene expression (log10 FPKM), red means up regulation and blue means down regulation. The gene cluster tree is on the left, the name of DEGs is on the right.

**Figure 7:**
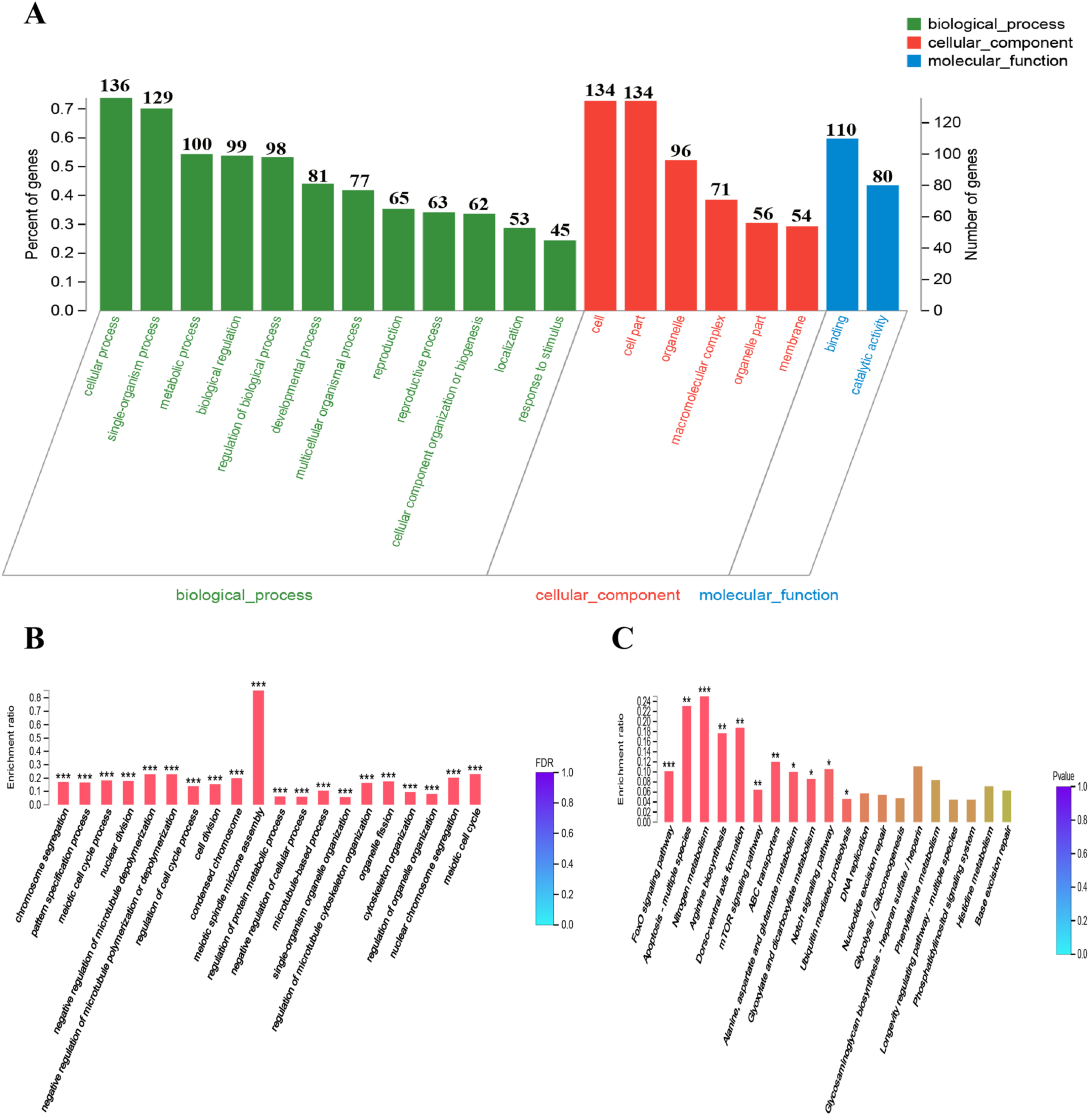
DEGs GO and KEGG enrichment analysis. (A) GO classification bar chart. The horizontal scale at the top indicates the number of gene classified to each sub-categories of GO terms; The horizontal scale at the bottom indicates the percentage of genes of each sub-categories to total genes which are successfully annotated by GO assignments. Sub-categories of biological processes (BP), cellular components (CC), and molecular function (MF) are displayed in detail on the *Y*-axis. (B) and (C) DEGs GO and KEGG enrichment histogram. The *x*-axis represents cluster and subcluster of GO terms or KEGG pathway enriched, and the *y*-axis represents enrichment ratio (Enrichment ratio = Sample number/Background number). The color gradient represents the value of FDR or *p* value. The “***” , “**” and “*” means that FDR (*p*-value) <0.001, FDR (*p*-value) < 0.01, and FDR (*p*-value) < 0.05, respectively.

To provide some insight into the effect on gene expression patterns when *anp-1* deleted, we performed transcriptome sequencing and cluster analysis of DEGs by distance calculation algorithm between N2 strain and RB804 strain. All sequencing data were submitted in NCBI and can be accessed in the Short Read Archive (SRA) under accession number PRJNA548230. Among the genes identified, 184 genes were differentially expressed with 164 genes downregulated and 20 genes upregulated between N2 group and RB804 group, using a false discovery rate ≤0.05 and a fold change ≥2 as significant cutoffs (Table S2). As shown in Fig. 6, the heatmap profile indicated DEGs among different groups. Of the DEGs, 184 genes were annotated into 47 sub-categories belonging to the following three main GO categories: biological process (BP), cellular component (CC) and molecular function (MF) (Fig. 7A). The BP sub-categories, CC sub-categories, and MF sub-categories were 22, 14 and 11, respectively. In BP sub-categories, the vast majority was related to cellular process, single-organism process, metabolic process, biological regulation, regulation of BP, developmental process, multicellular organismal process, reproduction, reproductive process, CC organization or biogenesis, localization and response to stimulus. In CC sub-categories, genes for cell, cell part, organelle, macromolecular complex, organelle part, and membrane were the top six. Among the MF sub-categories, the majority of the GO terms were grouped into binding, catalytic activity. The detailed information for the annotations was in Table S3.

GO enrichment analysis placed 184 DEGs into 1,062 functional categories: meiotic spindle midzone assembly was the largest cluster, followed by meiotic cell cycle, negative regulation of microtubule depolymerization, and negative regulation of microtubule polymerization or depolymerization, etc. (Fig. 7B). Based on KEGG pathway mapping, 85 DEGs were classified into the following 45 KEGG functional categories (e.g., FoxO signaling pathway, Apoptosis-multiple species, Nitrogen metabolism, Arginine biosynthesis, Dorso-ventral axis formation, mTOR signaling pathway). A summary of the findings are presented in Fig. 7C and Table S4. According to further KEGG analysis of the 164 down-regulated genes and 20 up-regulated genes between N2 strain and RB804 strain, the top sub functional categories were FoxO signaling pathway genes, mTOR signaling pathway genes, and ABC transporters, respectively. The detailed annotation for the results were summarized in Fig. S5 and Table S5.

### qRT-PCR assay

**Figure 8:**
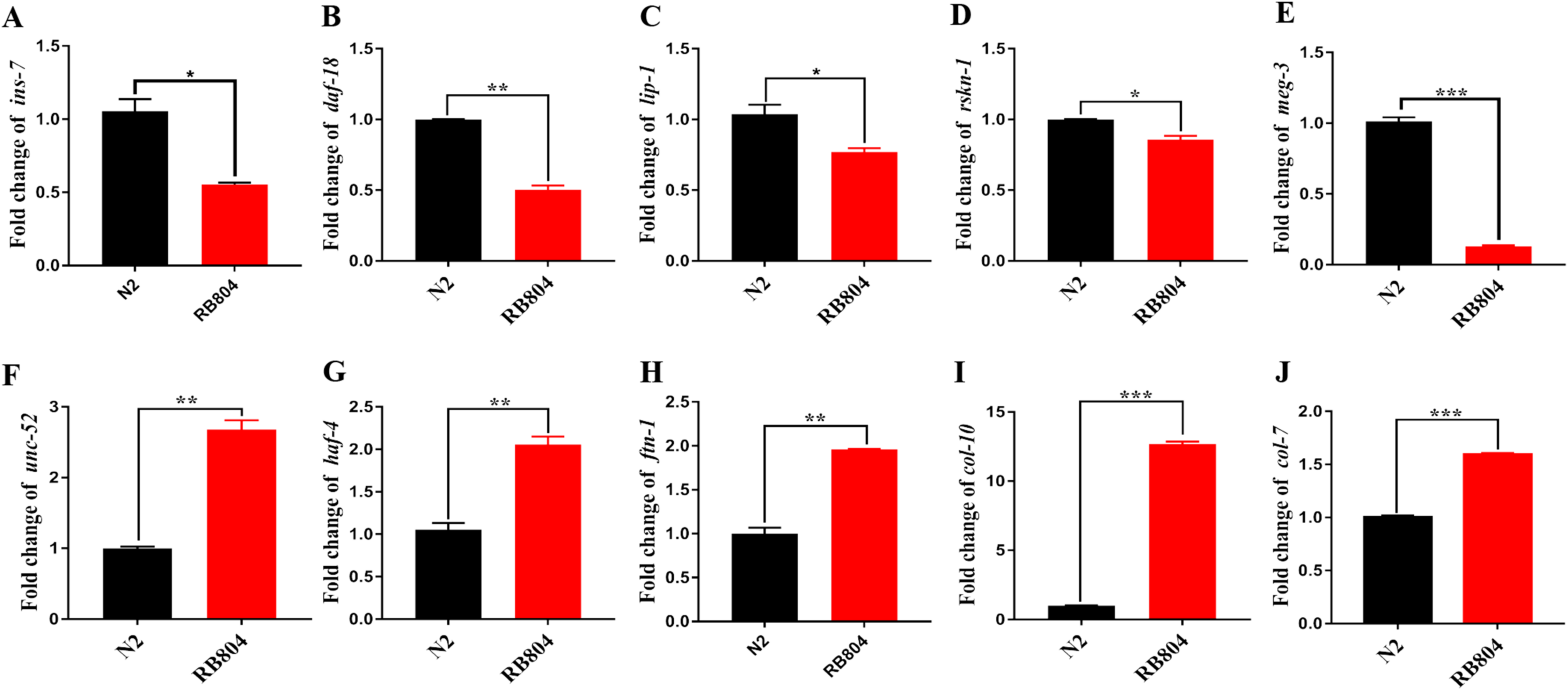
Expression patterns of DEGs between N2 strain and *anp-1* knockout strain verified by qRT-PCR. (A–E) DEGs down-regulated. (F–J) DEGs up-regulated. The ***,**, and * indicate statistical significance *p* < 0.001, *p* < 0.01 and *p* < 0.05, respectively.

To validate expression patterns of DEGs and to determine the potential roles of the genes in regulation pathway, we confirmed their differential expression patterns between wild type and *anp-1* knockout animals by qRT-PCR. Expression level of DEGs were consistent with those obtained by transcriptome sequencing results, confirming the accuracy of the transcriptome sequencing results reported in this study. Those genes, including *ins-7*, *daf-18*, *lip-1*, *rskn-1*, *meg-3*, *mex-1*, *lin-22*, *vit-2*, *gna-2*, *hop-1*, and *klp-5* were down-regulated, however, *unc-52*, *ftn-1*, *col-7*, *col-10*, *haf-4*, *haf-9*, *F49E2.5*, *dpy-1*, and *glt-7* were up-regulated (Fig. 8; Fig. S6). Based on the KEGG classified results, these down-regulated genes (*lip-1* and *rskn-1*) were classified as MAPK signaling pathway sub-cluster. The longevity regulating pathway components, *daf-18* and *ins-7* were down-regulated in RB804 group compared with N2 group. *Daf-18* and *cyb-1* belonging to FoxO signaling pathway sub-cluster were down-regulated in RB804 group (Table S6).

## Discussion

Aminopeptidases are exopeptidases that cleave amino acids from the N-terminus of protein or peptide substrates. They play a major role in regulating the balance between catabolism and anabolism in all living cells (Peer, 2011; Rawlings, Tolle & Barrett, 2004). In *C. elegans* and other nematodes, aminopeptidases are very important for growth, digestion, molting, and fertility (Joshua, 2001; Malagon et al., 2010; McKerrow et al., 2006; Page et al., 2014). The role of most M1 aminopeptidase orthologs in reproduction has been demonstrated in *C. elegans* (Althoff, Flick & Trzepacz, 2014). Previous studies have shown that PAM-1 and other M1 aminopeptidase orthologs, but not ANP-1, can retain their catalytic functions and are involved in the generation of progeny at multiple stages (Althoff, Flick & Trzepacz, 2014; Brooks, Hooper & Isaac, 2003). However, Our brood size analysis revealed that *anp-1* has affect on fertility in *C. elegans*. Furthermore, expression pattern analysis shows that ANP-1 is expressed throughout *C. elegans* tissues (Hunt-Newbury et al., 2007). We conjectured that ANP-1 not only play important roles in reproduction but also participate in other physiological processes in *C. elegans*.

First of all, we report the identification of ANP-1 in *C. elegans* along with sequence analysis and its functional expression and characterization. ANP-1 was determined to be a typical M1 aminopeptidase, sharing conserved motifs with aminopeptidases in other organisms including parasitic nematodes. A comparison with the amino acid sequences of other members of the M1 aminopeptidase family showed that general conservation was restricted to the N-terminal region, which is indispensable for the zinc ion coordination and enzyme catalytic functions of aminopeptidases (Luan et al., 2012), whereas the C-terminal portion was more divergent. Phylogenetic analysis revealed that ANP-1 and its orthologs (R03G8.4 and R03G8.6) assembled into a well-defined cluster distinguishable from other aminopeptidases in *C. elegans*, indicating the possibility that these aminopeptidases may play different roles in *C. elegans’* biological processes. The 3D structure of ANP-1 showed that Zn^2+^, a metal ion cofactor, was surrounded by histidines (366 and 400 anchoring residues) and glutamic acid (419 anchoring residue). The proximal glutamic acid is essential for the hydrolysis of peptide bonds and subsequent release of the substrate (Thompson, Govindaswami & Hersh, 2003). The APN (Aminopeptidase N, APN) exists in two forms, namely, membrane APN and soluble APN. N-terminus of APN is anchoring to cell membrane, and catalytic domain of APN is presenting outside the cell. APNs are sensitive to proteolysis and can be degraded into two parts, with MW ranging between 90 kDa and 45 kDa (Luan et al., 2012). We found that ANP-1 existed as a membrane protein in *C. elegans*, it might be degraded into two parts, the small portion of ANP-1 was larger than that of other APNs. The heterogeneity of ANP-1 is on the further processing. APNs are heavily glycosylated proteins (Peer, 2011), among which ANP-1 is one with four glycosylation residues. N-glycans play diverse biological roles in differentiation, development, embryogenesis, and inflammatory response (Lowe, 2002; Varki, 1993). In *C. elegans*, 304 proteins are identified as glycosylated proteins (Fan et al., 2005; Kaji et al., 2003). However, only a relatively small number of genes in glycobiology have been expressed and functionally characterized using RNAi (Schachter, 2004). Our results showed that glycosylated aminopeptidases including ANP-1 may participate in the development and aging of *C. elegans*.

In *C. elegans*, PAM-1 may play important roles in fertility-regulating through, or in collaboration with, the MAPK pathway. RNAi-mediated suppression of the aminopeptidase orthologs of PAM-1 decreases brood size of *C. elegans* (Althoff, Flick & Trzepacz, 2014*)*. It is clear that double-stranded RNA-triggered interference is an efficient approach to validate target gene function in a number of model organisms (Melnyk et al., 2011; Timmons & Fire, 1998; Wall & Shi, 2003). But it is becoming apparent that the pitfalls associated with the technique preclude its use as RNAi may induce off-target effects (Geldhof et al., 2007). However, our data were different with the conclusion of Althoff, Flick & Trzepacz (2014). In the present study, *anp-1* deletion remarkably decreased the brood size compared with normal worms, and DEGs were identified in MAPK pathway. Moreover, qRT-PCR analysis validated the expression patterns of DEGs (such as *lip-1* and *rskn-1*). So, we assume that ANP-1 participates in fertility-governing function in collaboration with the MAPK pathway. However, more efforts are needed to reveal the molecular mechanism that ANP-1 mediated fertility regulation.

Proteases encompass a broad class of hydrolytic enzymes that play essential roles in cell development and digestive processes (McKerrow et al., 2006; Skinner-Adams et al., 2010). PAM-1, a homolog of ANP-1, is expressed in the intestine and may function in the process of peptides absorbed by the intestinal cells following the breakdown of proteins in the gut lumen (Brooks, Hooper & Isaac, 2003). Homologs of aminopeptidase P and LAP from *C. elegans* have been proposed to have the same function (Joshua, 2001). As we mentioned above, ANP-1 is mainly expressed in intestine of *C. elegans* (Fig. S7), thus, we speculate that mutation of *anp-1* can affect peptide digestion and absorption in *C. elegans*. However, further studies are needed to validate the mechanism of ANP-1 mediated reduction in body size.

In our study, *anp-1* knockout significantly decreased the life-span of *C. elegans* and transcriptome sequencing analysis showed that *anp-1* mutation resulted in expression changes of components belonging to FoxO signaling pathway, such as *daf-18* and *cyb-1*. In line with this result, qRT-PCR assay indicated the expression of *daf-18* and *cyb-1* in *anp-1* deletion strain were dramatically lower than in normal worms. Previously studies have demonstrated the function of the FoxO (Forkhead TFs) pathway in mediating longevity (Samuelson, Carr & Ruvkun, 2007). In *C*. *elegans*, *daf-18* was identified as the *C. elegans* PTEN ortholog, DAF-18/PTEN participates in life-span regulation by by mediating DAF-16/ FoxO phosphorylation. *Daf-18* null mutant induced shortened life span phonetype (Murphy & Hu, 2013). According to our results, it is reasonable to speculate that knockout of *anp-1* can decrease the life-span by affecting the expression of DAF-16/ FoxO pathway molecules, for example, *daf-18*. However, insulin-like peptides (ILPs) can regulate longevity in *C. elegans* (Murphy & Hu, 2013), ILPs like *ins-7* have been studied in some depth, but the biological functions of the majority of ILPs remain unknown (Murphy & Hu, 2013). We found *anp-1* mutation resulted in typically expression decrease in *ins-7*. Although our results indicated aminopeptidase ANP-1 may have a vital physiological function in ILPs, with such diverse mechanisms of genetically regulated longevity in *C. elegans*, it is clear that we need to further investigate genetic mechanisms to fully understand the process of aging (Finch & Austad, 2001). However, as the interaction between aminopeptidases and longevity may be still be underestimated, we are conducting further studies to examine it.

## Conclusions

In summary, loss-of-function of the aminopeptidase gene *anp-1* induced shortened lifespan, decreased body size and brood size in *C. elegans*. Even though many studies focus on the functions of aminopeptidases in *C. elegans*, this is the first report on the role of the aminopeptidases in lifespan and body size. Our data suggest that aminopeptidase ANP-1 participates in development and lifespan regulation in *C. elegans*. This study laid the foundation for further research to validate the physiological functions of aminopeptidases in *C. elegans*. Accordingly, our future studies will focus on gene regulation mechanisms associated with aminopeptidases.

## Supplemental Information

10.7717/peerj.7944/supp-1 Supplemental Information 1 Complete nucleotide and deduced amino acid sequence of *anp-1*. The deduced amino acid sequences are below the nucleotide sequences. The initiation codon (ATG) and stop codon (TAA) are in underlined italics.

10.7717/peerj.7944/supp-2 Supplemental Information 2 Sequence alignment analysis of the M1 aminopeptidase family. (A) Multiple sequence alignments of M1 aminopeptidase family members. The conserved zinc-binding motif (HEXXH-X18-H) and a GEMAN motif are shown in the box. (B) Predicted functional domains of ANP-1. The zinc-binding residues are denoted by the long lines. The glycosylation sites are located at residue 142, 341, 544 and 567.

10.7717/peerj.7944/supp-3 Supplemental Information 3 Secondary structure prediction of ANP-1 based on the 5lg6.1.A . α means α-α-helice; β represents β-pleated sheet; η indicates random coil.

10.7717/peerj.7944/supp-4 Supplemental Information 4 Representative images of animal body length of N2 strain and RB804 strain. The bar represents 200 μm.

10.7717/peerj.7944/supp-5 Supplemental Information 5 KEGG enrichment analysis of 164 down-regulated and 20 up-regulated genes. The x- axis represents cluster and subcluster of KEGG pathway enriched, and the y -axis represents enrichment ratio (Enrichment ratio =Sample number/Background number). The color gradient represents the value of *p* value. The “***” , “**” and “*” means that *p* value <0.001, *p* value <0.01, and *p* value <0.05, respectively.

10.7717/peerj.7944/supp-6 Supplemental Information 6 qTR-PCR analysis of expression patterns of DEGs. The “***” , “**” and “*” means that *p* value <0.001, *p* value <0.01, and *p* value <0.05, respectively.

10.7717/peerj.7944/supp-7 Supplemental Information 7 The expression pattern of ANP-1 identified using polyclonal antibody against rANP-1.

10.7717/peerj.7944/supp-8 Supplemental Information 8 Primers used in this study.

10.7717/peerj.7944/supp-9 Supplemental Information 9 DEGs identified between N2 group and RB804 group.

10.7717/peerj.7944/supp-10 Supplemental Information 10 GO annotations of 184 DEGs.

10.7717/peerj.7944/supp-11 Supplemental Information 11 KEGG pathway analysis of 184 DEGs.

10.7717/peerj.7944/supp-12 Supplemental Information 12 Supplementary Table 5 KEGG enrichment analysis of 164 down-regulated DEGs and 20 up-regulated DEGs.

10.7717/peerj.7944/supp-13 Supplemental Information 13 KEGG enrichment analysis of down-regulated or up-regulated DEGs.

10.7717/peerj.7944/supp-14 Supplemental Information 14 Full length of gels and bolts.

## Additional Information and Declarations

### Data Availability

The following information was supplied regarding data availability: All sequencing data are available in the SRA: PRJNA548230.

## References

[ref-1] Althoff MJ, Flick K, Trzepacz C (2014). Collaboration within the M1 aminopeptidase family promotes reproductive success in *Caenorhabditis elegans*. Development Genes and Evolution.

[ref-2] Brooks DR, Hooper NM, Isaac RE (2003). The *Caenorhabditis elegans* orthologue of mammalian puromycin-sensitive aminopeptidase has roles in embryogenesis and reproduction. Journal of Biological Chemistry.

[ref-3] Constam DB, Tobler AR, Rensing-Ehl A, Kemler I, Hersh LB, Fontana A (1995). Puromycin-sensitive aminopeptidase: sequence analysis, expression, and functional characterization. Journal of Biological Chemistry.

[ref-4] Dereeper A, Guignon V, Blanc G, Audic S, Buffet S, Chevenet F, Dufayard JF, Guindon S, Lefort V, Lescot M, Claverie JM, Gascuel O (2008). Phylogeny.fr: robust phylogenetic analysis for the non-specialist. Nucleic Acids Research.

[ref-5] Drinkwater N, Lee J, Yang W, Malcolm TR, McGowan S (2017). M1 aminopeptidases as drug targets: broad applications or therapeutic niche?. FEBS Journal.

[ref-6] Fan X, She YM, Bagshaw RD, Callahan JW, Schachter H, Mahuran DJ (2005). Identification of the hydrophobic glycoproteins of *Caenorhabditis elegans*. Glycobiology.

[ref-7] Finch CE, Austad SN (2001). History and prospects: symposium on organisms with slow aging. Experimental Gerontology.

[ref-8] Geldhof P, Visser A, Clark D, Saunders G, Britton C, Gilleard J, Berriman M, Knox D (2007). RNA interference in parasitic helminths: current situation, potential pitfalls and future prospects. Parasitology.

[ref-9] Hersh LB (1981). Inhibition of aminopeptidase and acetylcholinesterase by puromycin and puromycin analogs. Journal of Neurochemistry.

[ref-10] Hui KS, Wang YJ, Lajtha A (1983). Purification and characterization of an enkephalin aminopeptidase from rat brain membranes. Biochemistry.

[ref-11] Hunt-Newbury R, Viveiros R, Johnsen R, Mah A, Anastas D, Fang L, Halfnight E, Lee D, Lin J, Lorch A, McKay S, Okada HM, Pan J, Schulz AK, Tu D, Wong K, Zhao Z, Alexeyenko A, Burglin T, Sonnhammer E, Schnabel R, Jones SJ, Marra MA, Baillie DL, Moerman DG (2007). High-throughput in vivo analysis of gene expression in *Caenorhabditis elegans*. PLOS Biology.

[ref-12] Iturrioz X, Rozenfeld R, Michaud A, Corvol P, Llorens-Cortes C (2001). Study of asparagine 353 in aminopeptidase A: characterization of a novel motif (GXMEN) implicated in exopeptidase specificity of monozinc aminopeptidases. Biochemistry.

[ref-13] Joshua GWP (2001). Functional analysis of leucine aminopeptidase in *Caenorhabditis elegans*. Molecular and Biochemical Parasitology.

[ref-14] Kaji H, Saito H, Yamauchi Y, Shinkawa T, Taoka M, Hirabayashi J, Kasai K, Takahashi N, Isobe T (2003). Lectin affinity capture, isotope-coded tagging and mass spectrometry to identify N-linked glycoproteins. Nature Biotechnology.

[ref-15] Kamath RS, Fraser AG, Dong Y, Poulin G, Durbin R, Gotta M, Kanapin A, Le Bot N, Moreno S, Sohrmann M, Welchman DP, Zipperlen P, Ahringer J (2003). Systematic functional analysis of the *Caenorhabditis elegans* genome using RNAi. Nature.

[ref-16] Larkin MA, Blackshields G, Brown NP, Chenna R, McGettigan PA, McWilliam H, Valentin F, Wallace IM, Wilm A, Lopez R, Thompson JD, Gibson TJ, Higgins DG (2007). Clustal W and Clustal X version 2.0. Bioinformatics.

[ref-17] Laustsen PG, Vang S, Kristensen T (2001). Mutational analysis of the active site of human insulin-regulated aminopeptidase. European Journal of Biochemistry.

[ref-18] Lezzerini M, Van De Ven K, Veerman M, Brul S, Budovskaya YV (2015). Specific RNA interference in *Caenorhabditis elegans* by ingested dsRNA expressed in *Bacillus subtilis*. PLOS ONE.

[ref-19] Li B, Dewey CN (2011). RSEM: accurate transcript quantification from RNA-Seq data with or without a reference genome. BMC Bioinformatics.

[ref-20] Liang J, Xiong S, Savage-Dunn C (2013). Using RNA-mediated interference feeding strategy to screen for genes involved in body size regulation in the nematode *C. elegans*. Journal of Visualized Experiments.

[ref-21] Lowe JB (2002). Glycosylation in the control of selectin counter-receptor structure and function. Immunological Reviews.

[ref-22] Luan Y, Ma C, Wang Y, Fang H, Xu W (2012). The characteristics, functions and inhibitors of three aminopeptidases belonging to the m1 family. Current Protein & Peptide Science.

[ref-23] Lyczak R, Zweier L, Group T, Murrow MA, Snyder C, Kulovitz L, Beatty A, Smith K, Bowerman B (2006). The puromycin-sensitive aminopeptidase PAM-1 is required for meiotic exit and anteroposterior polarity in the one-cell *Caenorhabditis elegans* embryo. Development.

[ref-24] Malagon D, Diaz-Lopez M, Benitez R, Adroher FJ (2010). Cathepsin B- and L-like cysteine protease activities during the in vitro development of *Hysterothylacium aduncum* (Nematoda: anisakidae), a worldwide fish parasite. Parasitology International.

[ref-25] Matsui M, Fowler JH, Walling LL (2006). Leucine aminopeptidases: diversity in structure and function. Biological Chemistry.

[ref-26] McKerrow JH, Caffrey C, Kelly B, Loke P, Sajid M (2006). Proteases in parasitic diseases. Annual Review of Pathology: Mechanisms of Disease.

[ref-27] McLellan S, Dyer SH, Rodriguez G, Hersh LB (1988). Studies on the tissue distribution of the puromycin-sensitive enkephalin-degrading aminopeptidases. Journal of Neurochemistry.

[ref-28] Melnyk CW, Molnar A, Bassett A, Baulcombe DC (2011). Mobile 24 nt small RNAs direct transcriptional gene silencing in the root meristems of *Arabidopsis thaliana*. Current Biology.

[ref-29] Mucha A, Drag M, Dalton JP, Kafarski P (2010). Metallo-aminopeptidase inhibitors. Biochimie.

[ref-30] Munkhjargal T, Ishizaki T, Guswanto A, Takemae H, Yokoyama N, Igarashi I (2016). Molecular and biochemical characterization of methionine aminopeptidase of *Babesia bovis* as a potent drug target. Veterinary Parasitology.

[ref-31] Murphy CT, Hu PJ (2013). Insulin/insulin-like growth factor signaling in *C. elegans*. WormBook: The Online Review of C. elegans Biology.

[ref-32] Osada T, Sakaki Y, Takeuchi T (1999). Puromycin-sensitive aminopeptidase gene (*Psa*) maps to mouse chromosome 11. Genomics.

[ref-33] Page AP, Stepek G, Winter AD, Pertab D (2014). Enzymology of the nematode cuticle: a potential drug target?. International Journal for Parasitology: Drugs and Drug Resistance.

[ref-34] Peer WA (2011). The role of multifunctional M1 metallopeptidases in cell cycle progression. Annals of Botany.

[ref-35] Pradillo M, Lopez E, Romero C, Sanchez-Moran E, Cunado N, Santos JL (2007). An analysis of univalent segregation in meiotic mutants of *Arabidopsis thaliana*: a possible role for synaptonemal complex. Genetics.

[ref-36] Rawlings ND, Barrett AJ, Bateman A (2012). MEROPS: the database of proteolytic enzymes, their substrates and inhibitors. Nucleic Acids Research.

[ref-37] Rawlings ND, Tolle DP, Barrett AJ (2004). Evolutionary families of peptidase inhibitors. Biochemical Journal.

[ref-38] Redmond DL, Geldhof P, Knox DP (2004). Evaluation of *Caenorhabditis elegans* glycoproteins as protective immunogens against *Haemonchus contortus* challenge in sheep. International Journal for Parasitology.

[ref-39] Roberts B, Antonopoulos A, Haslam SM, Dicker AJ, McNeilly TN, Johnston SL, Dell A, Knox DP, Britton C (2013). Novel expression of *Haemonchus contortus* vaccine candidate aminopeptidase H11 using the free-living nematode *Caenorhabditis elegans*. Veterinary Research.

[ref-40] Robinson MD, McCarthy DJ, Smyth GK (2010). edgeR: a bioconductor package for differential expression analysis of digital gene expression data. Bioinformatics.

[ref-56] Samuelson AV, Carr CE, Ruvkun G (2007). Gene activities that mediate increased life span of *C. elegans* insulin-like signaling mutants. Genes & Development.

[ref-41] Sanchez-Moran E, Jones GH, Franklin FCH, Santos JL (2004). A puromycin-sensitive aminopeptidase is essential for meiosis in *Arabidopsis thaliana*. Plant Cell.

[ref-42] Schachter H (2004). Protein glycosylation lessons from *Caenorhabditis elegans*. Current Opinion in Structural Biology.

[ref-43] Schulz C, Perezgasga L, Fuller MT (2001). Genetic analysis of dPsa, the drosophila orthologue of puromycin-sensitive aminopeptidase, suggests redundancy of aminopeptidases. Development Genes and Evolution.

[ref-44] Skinner-Adams TS, Stack CM, Trenholme KR, Brown CL, Grembecka J, Lowther J, Mucha A, Drag M, Kafarski P, McGowan S, Whisstock JC, Gardiner DL, Dalton JP (2010). Plasmodium falciparum neutral aminopeptidases: new targets for anti-malarials. Trends in Biochemical Sciences.

[ref-45] Sonnichsen B, Koski LB, Walsh A, Marschall P, Neumann B, Brehm M, Alleaume AM, Artelt J, Bettencourt P, Cassin E, Hewitson M, Holz C, Khan M, Lazik S, Martin C, Nitzsche B, Ruer M, Stamford J, Winzi M, Heinkel R, Roder M, Finell J, Hantsch H, Jones SJ, Jones M, Piano F, Gunsalus KC, Oegema K, Gonczy P, Coulson A, Hyman AA, Echeverri CJ (2005). Full-genome RNAi profiling of early embryogenesis in *Caenorhabditis elegans*. Nature.

[ref-46] Stiernagle T (2006). Maintenance of *C. elegans*. WormBook: The Online Review of C. elegans Biology.

[ref-47] Thompson MW, Govindaswami M, Hersh LB (2003). Mutation of active site residues of the puromycin-sensitive aminopeptidase: conversion of the enzyme into a catalytically inactive binding protein. Archives of Biochemistry and Biophysics.

[ref-48] Timmons L, Fire A (1998). Specific interference by ingested dsRNA. Nature.

[ref-49] Tobler AR, Constam DB, Schmitt-Graff A, Malipiero U, Schlapbach R, Fontana A (1997). Cloning of the human puromycin-sensitive aminopeptidase and evidence for expression in neurons. Journal of Neurochemistry.

[ref-50] Trapnell C, Pachter L, Salzberg SL (2009). TopHat: discovering splice junctions with RNA-Seq. Bioinformatics.

[ref-51] Varki A (1993). Biological roles of oligosaccharides: all of the theories are correct. Glycobiology.

[ref-52] Wall NR, Shi Y (2003). Small RNA: can RNA interference be exploited for therapy?. Lancet.

[ref-53] Xie C, Mao X, Huang J, Ding Y, Wu J, Dong S, Kong L, Gao G, Li CY, Wei L (2011). KOBAS 2.0: a web server for annotation and identification of enriched pathways and diseases. Nucleic Acids Research.

[ref-54] Yu L, Yan X, Ye C, Zhao H, Chen X, Hu F, Li H (2015). Bacterial respiration and growth rates affect the feeding preferences, brood size and lifespan of *Caenorhabditis elegans*. PLOS ONE.

[ref-55] Zhou QJ, Yang Y, Guo XL, Duan LJ, Chen XQ, Yan BL, Zhang HL, Du AF (2014). Expression of *Caenorhabditis elegans*-expressed trans-HPS, partial aminopeptidase H11 from *Haemonchus contortus*. Experimental Parasitology.

