# Supplementary figures and images for: Characterization of aminopeptidase encoding gene anp-1 and its association with development in Caenorhabditis elegans
Source: PeerJ. 2019 Nov 12;7:e7944. doi: 10.7717/peerj.7944 (PMC6857582; doi:10.7717/peerj.7944)

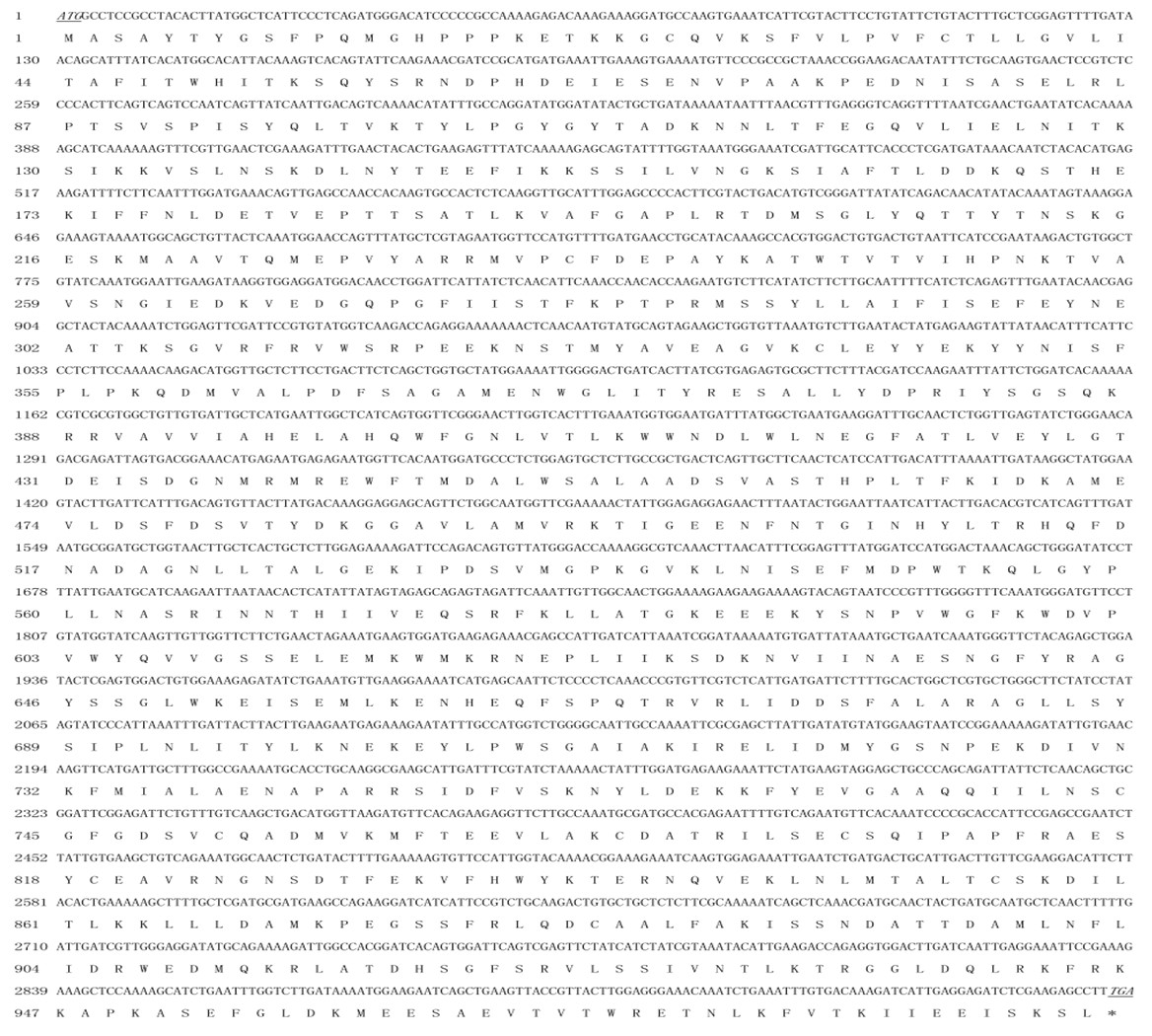

Supplement: Supplemental Information 1 — The deduced amino acid sequences are below the nucleotide sequences. The initiation codon (ATG) and stop codon (TAA) are in underlined italics. [file peerj-07-7944-s001.jpg]

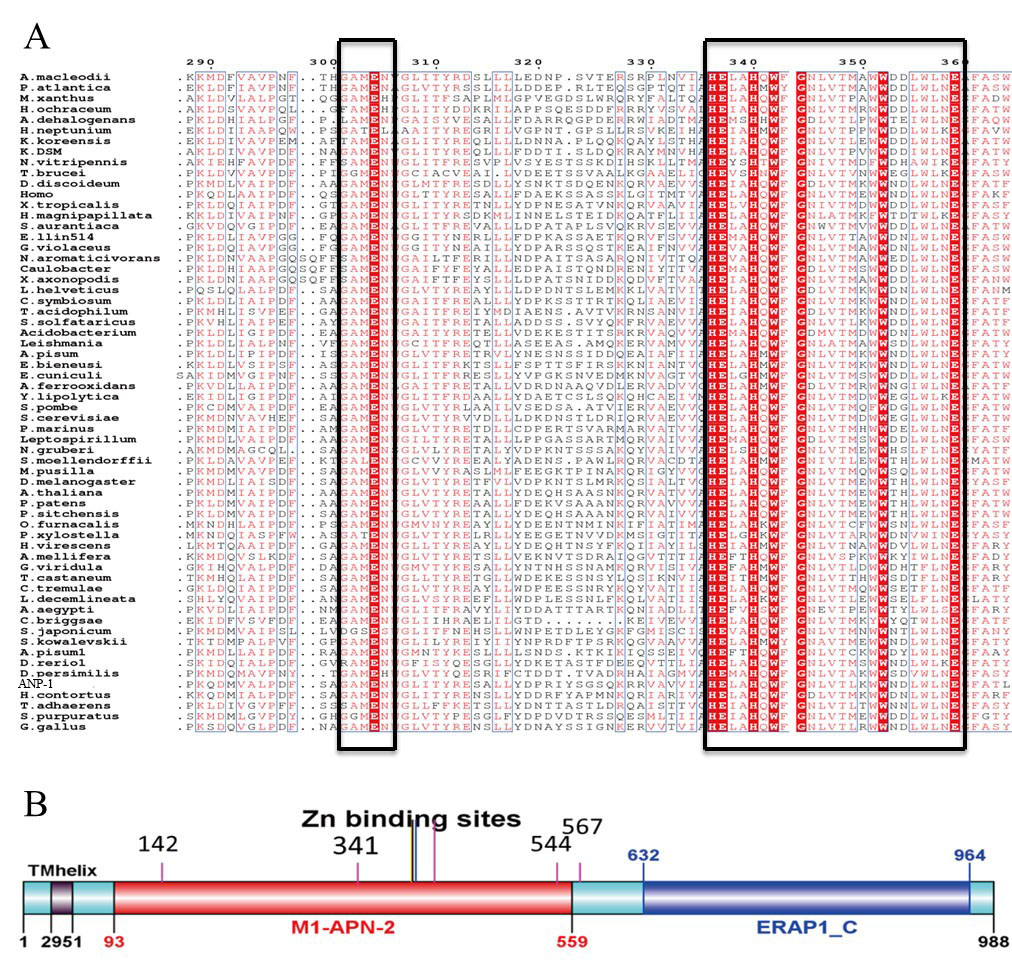

Supplement: Supplemental Information 2 — (A) Multiple sequence alignments of M1 aminopeptidase family members. The conserved zinc-binding motif (HEXXH-X18-H) and a GEMAN motif are shown in the box. (B) Predicted functional domains of ANP-1. The zinc-binding residues are denoted by the long lines. The glycosylation sites are located at residue 142, 341, 544 and 567. [file peerj-07-7944-s002.jpg]

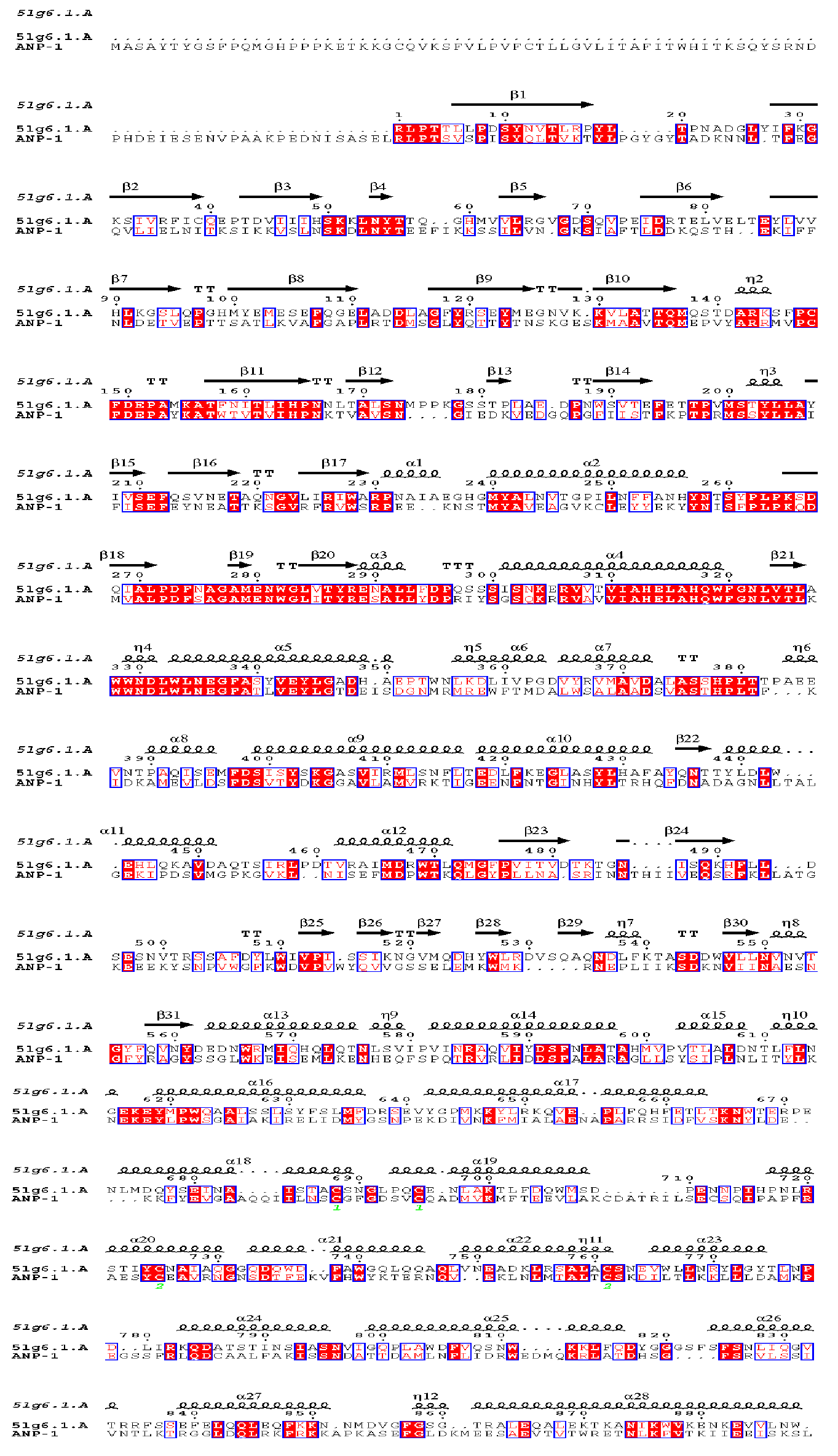

Supplement: Supplemental Information 3 — α means α-α-helice; β represents β-pleated sheet; η indicates random coil. [file peerj-07-7944-s003.png]

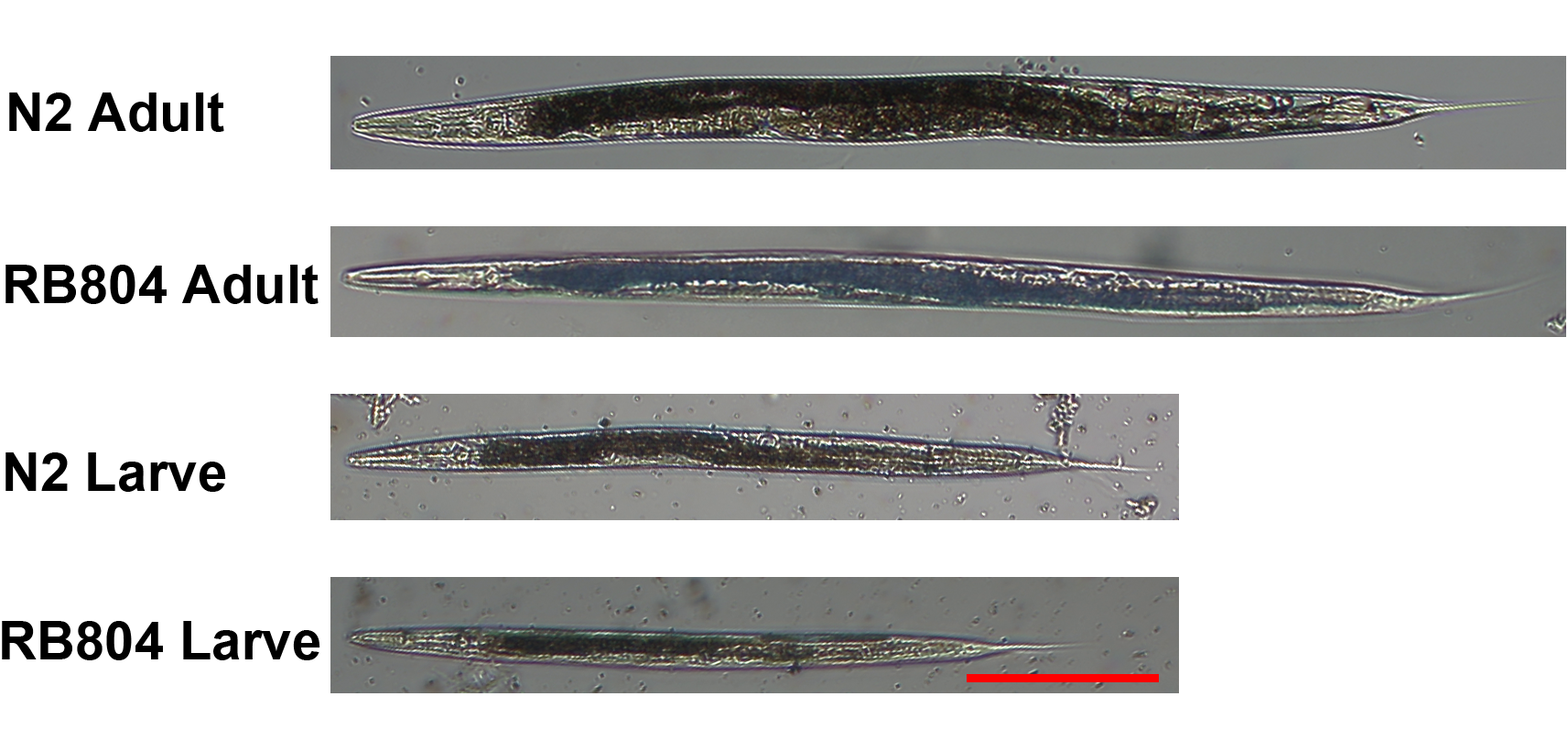

Supplement: Supplemental Information 4 — The bar represents 200 μm. [file peerj-07-7944-s004.png]

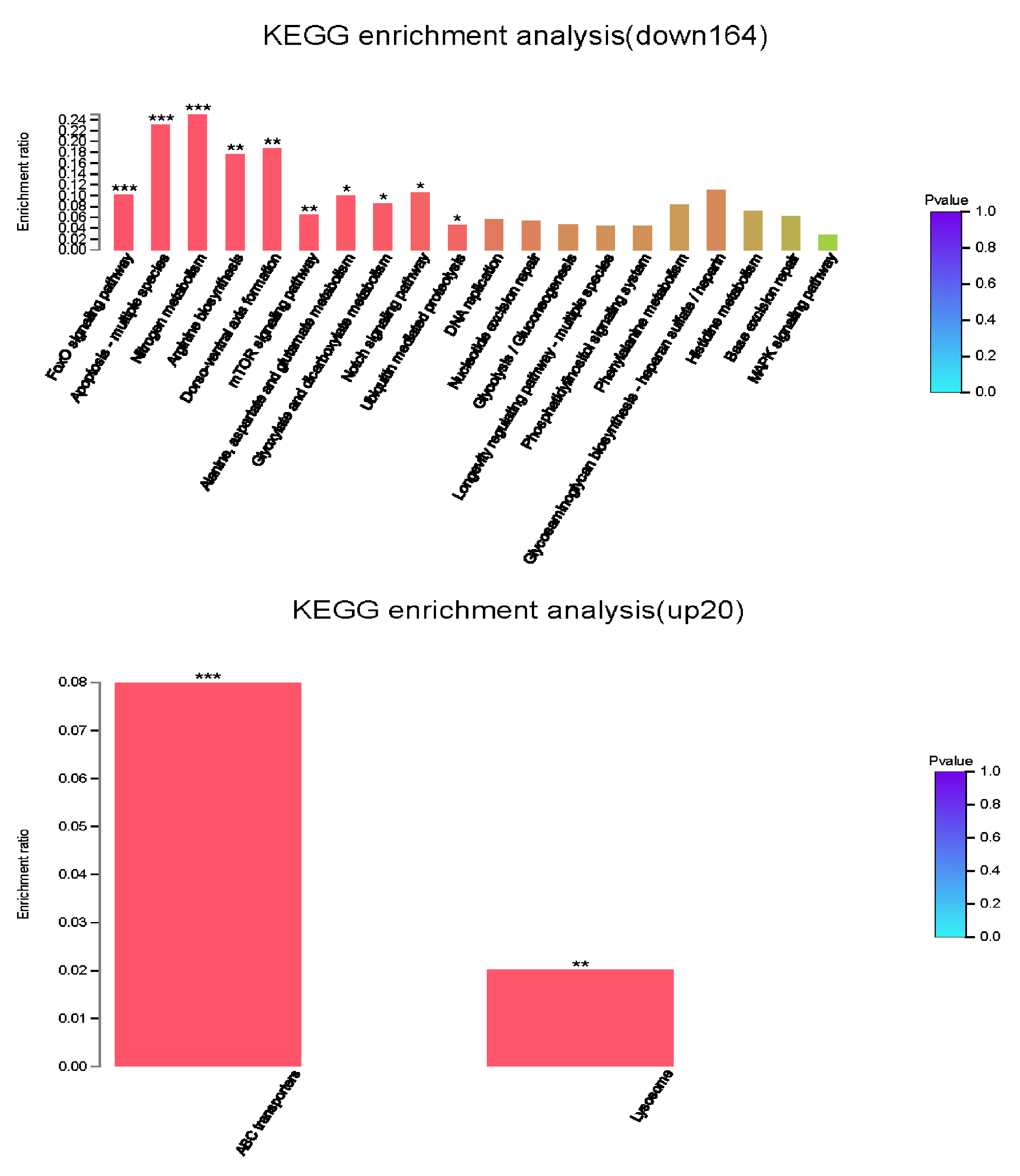

Supplement: Supplemental Information 5 — The x- axis represents cluster and subcluster of KEGG pathway enriched, and the y -axis represents enrichment ratio (Enrichment ratio =Sample number/Background number). The color gradient represents the value of p value. The “***” , “**” and “*” means that p value <0.001, p value <0.01, and p value <0.05, respectively. [file peerj-07-7944-s005.png]

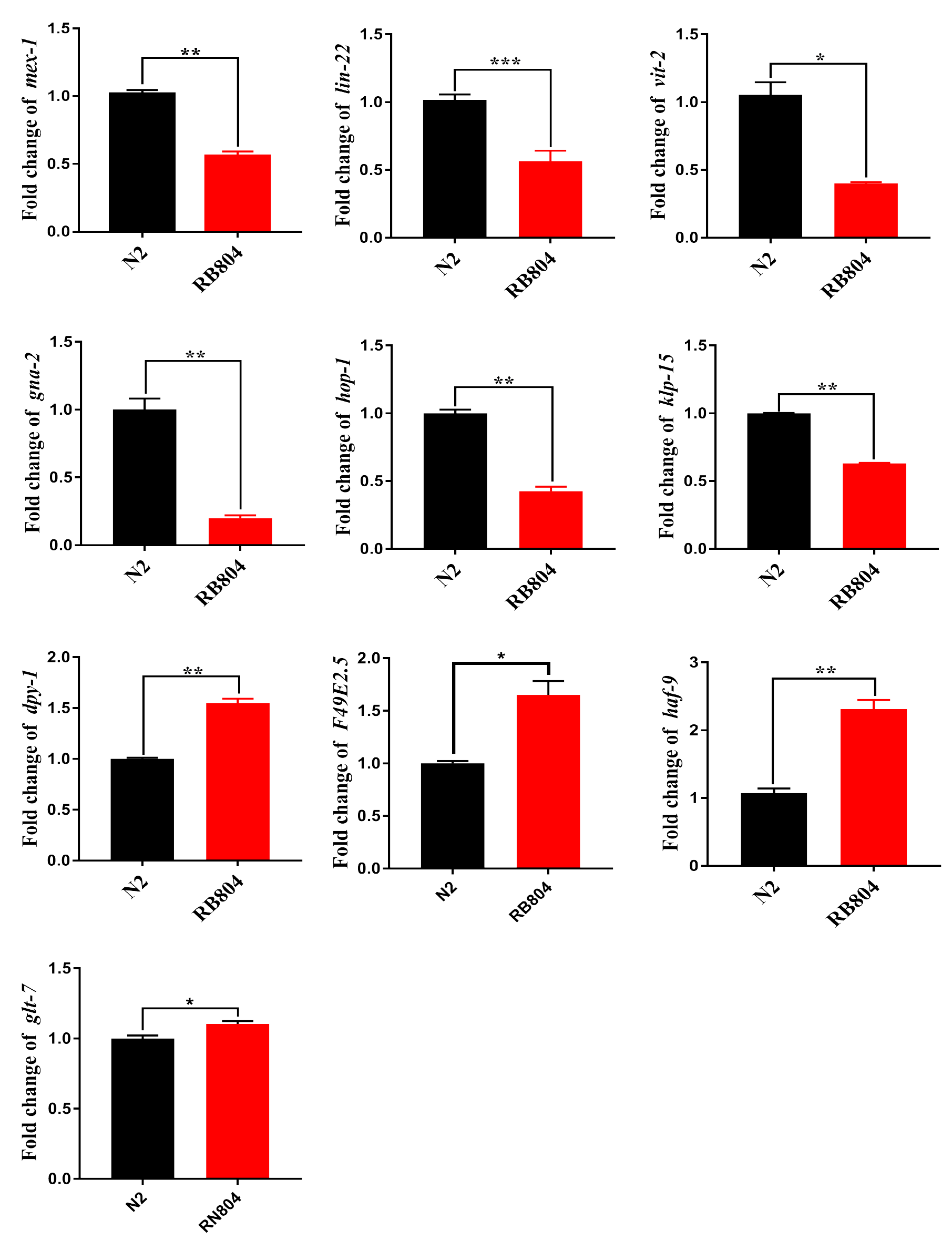

Supplement: Supplemental Information 6 — The “***” , “**” and “*” means that p value <0.001, p value <0.01, and p value <0.05, respectively. [file peerj-07-7944-s006.png]

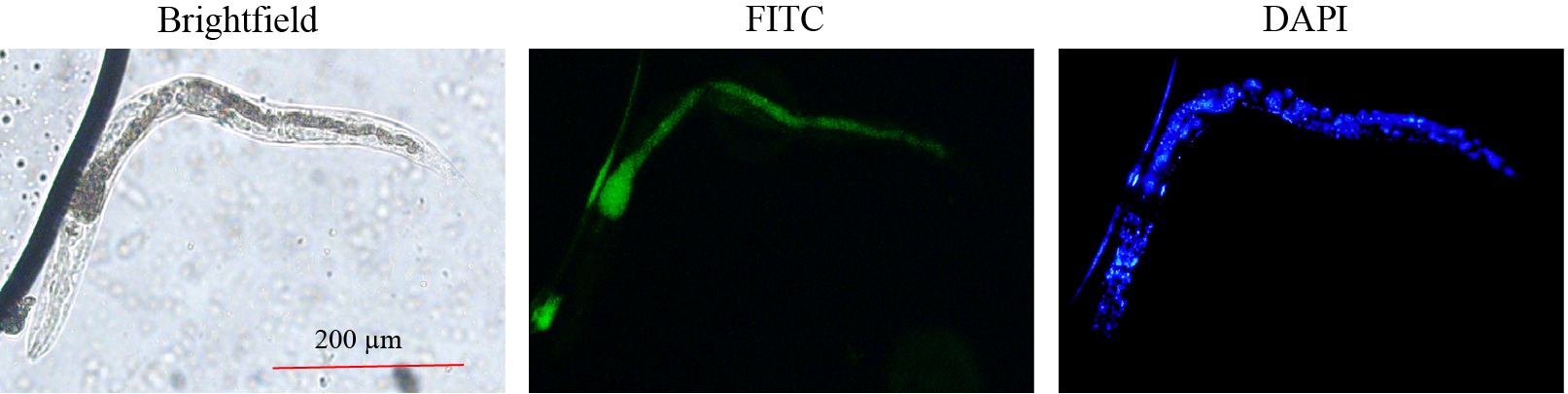

Supplement: Supplemental Information 7 [file peerj-07-7944-s007.png]
